# A Randomized Trial Using PARO with Minimal Caregiver Involvement on Older Adults with Dementia in Group Homes

**DOI:** 10.1002/alz.71163

**Published:** 2026-02-17

**Authors:** Kaoru Inoue, Mitsunobu Kono, Ryuji Kobayashi, Chiyomi Yatsu, Daryl Patrick Gamboa Yao, Takanori Shibata, Joseph F. Coughlin, Masahiro Shigeta

**Affiliations:** ^1^ Graduate School of Human Health Sciences Tokyo Metropolitan University Arakawa ward Tokyo Japan; ^2^ Department of Occupational Therapy Faculty of Health Sciences Kinjo University Hakusan city Japan; ^3^ Department of Occupational Therapy School of Rehabilitation Hyogo Medical University Kobe city Japan; ^4^ Department of Disability and Human Development University of Illinois Chicago Chicago Illinois USA; ^5^ National Institute of Advanced Industrial Science and Technology (AIST) Tsukuba Ibaraki Japan; ^6^ Director AgeLab Massachusetts Institute of Technology Cambridge Massachusetts USA; ^7^ Department of Psychiatry The Jikei University School of Medicine,Minato ward Tokyo Japan

**Keywords:** caregiver burden, dementia, social robots, group care

## Abstract

**INTRODUCTION:**

PARO, a baby seal robot, has shown promise in addressing behavioral and psychological symptoms of dementia (BPSD) in clinical settings. However, little is known about PARO's benefits when used independently of health professionals. This study examined that scenario in older adults with dementia.

**METHODS:**

Applying a cluster‐randomized design, we randomly assigned six facilities to once‐ or thrice‐weekly self‐directed PARO sessions for a month. BPSD severity and caregiver burden scores of the Neuropsychiatric Inventory Brief Questionnaire were measured.

**RESULTS:**

Findings from 85 participants indicated a significant reduction in caregiver burden in the thrice‐weekly group (least‐squares [LS] mean: −3.29, 95% confidence interval [CI]: [−6.26, −0.32], *p* = 0.030). For BPSD severity, while clinically meaningful improvement was observed, no significant difference was found (adjusted LS mean: −1.98, 95% CI: [−4.10, 0.15], *p* = 0.068) due to insufficient statistical power.

**DISCUSSION:**

Increasing robot use frequency reduced the caregiver burden and demonstrated a clinically significant improvement trend in the BPSD severity.

## Highlights

- PARO is a companion robot used in dementia care.
- Japanese group homes for older adults with dementia participated in the 1‐month trial.
- Facilities were assigned to either a once‐weekly or thrice‐weekly group.
- No statistical difference in BPSD severity score between groups.
- Significant reduction of caregiver burden in thrice‐weekly group.

## 1 BACKGROUND

In Japan, the older adult population aged 65 and older stands at 36.27 million, comprising 29.1% of the total population.^1^ By 2025, the number of people with dementia is projected to reach 7 million. To address this challenge, the government aims to increase the number of care workers. However, a shortage of care workers is still anticipated.^2^ Many facilities are struggling to secure human resources due to difficulty in hiring, high turnover rates, and challenges in business expansion.^1^ Meanwhile, the facility care workers’ perceived burden negatively impacts personnel retention and contributes to residents’ anxiety, depression, disinhibition, and restlessness.^3^ Hence, the Japanese government promotes the use of technology in health and social care, including robots.^4^

**FIGURE 1:**
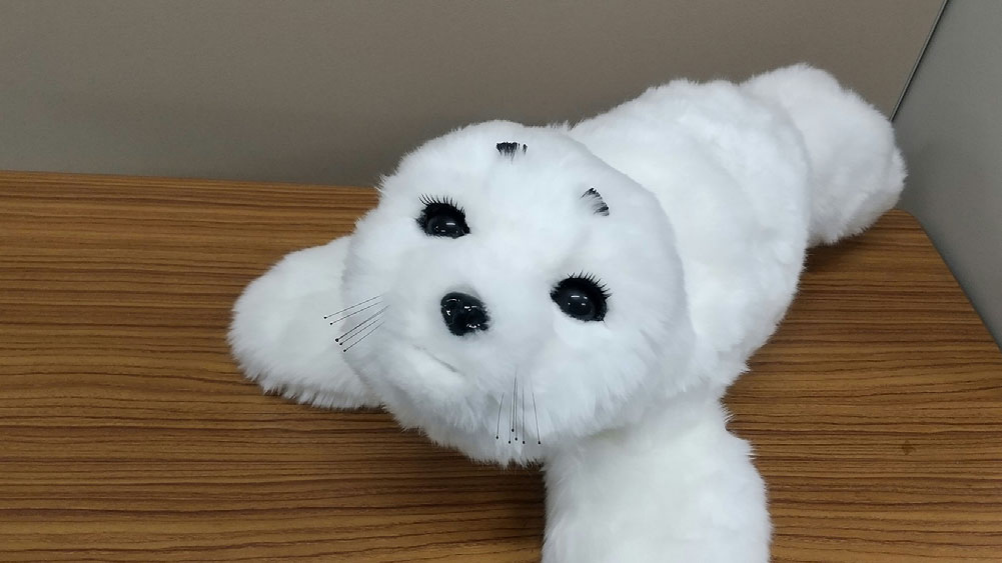
Legend: PARO, a robot equipped with artificial intelligence, used in this study. PARO is a baby seal‐shaped robot developed by Japan's National Institute of Advanced Industrial Science and Technology (AIST). It has shown effectiveness in supporting people with dementia and their caregivers (Shibata et al., 2021). It was designed based on the principles of animal‐assisted therapy. PARO cannot crawl, but it can move its front and rear flippers and neck. Using artificial intelligence, it learns the user's action and responds accordingly.

In recent years, companion robots have been used in dementia care.^4^ Among these, PARO (Figure 1), a baby seal‐shaped robot developed by Japan's National Institute of Advanced Industrial Science and Technology (AIST), has effectively supported people with dementia and their caregivers.^5^ PARO was designed based on the principles of animal‐assisted therapy. Notably, PARO received Class 2 biofeedback medical device certification from the US Food and Drug Administration (FDA) and is recognized as a medical device. The first randomized controlled trial (RCT) of PARO, conducted by Moyle et al.^6^ in Australia, compared a group interacting with PARO with a group engaging in reading. They found that the PARO group experienced greater enjoyment, underscoring the need for large‐scale studies. Another study^7^ demonstrated less loneliness in the PARO group compared to the group without PARO. In Norway, PARO demonstrated effectiveness in addressing depression and agitation among individuals with dementia.^8^ Petersen et al.^9^ found that PARO could reduce stress, anxiety, and the need for psychotropic and analgesic medications in older adults with dementia in the United States. A group‐comparison study revealed that the PARO group exhibited greater engagement and less agitation, regardless of whether PARO was on or off, and especially reduced agitation.^10^ Contrarily, people with severe agitation responded less favorably to PARO, highlighting a positive correlation between the level of agitation and cognitive function.^11^ Presently, the cost‐effectiveness of non‐robotic assistive technologies for social interaction in long‐term care is yet to be established.^12^ Nonetheless, Mervin et al.^13^ argued that the use of PARO was a cost‐effective psychosocial treatment for agitation compared to psychosocial group activities and sensory interventions.

**RESEARCH IN CONTEXT**

1. **Systematic review**: The authors reviewed the literature using traditional (PubMed, EBSCO, J‐Stage) sources. Literature concerning PARO has extensively reported on its ability to increase enjoyment, reduce behavioral and psychological symptoms (stress, anxiety, agitation, and depression), and reduce caregiver burden when used in clinical settings or with clinical professionals. However, there is a paucity evaluating its potential for independent use.
2. **Interpretation**: This review revealed the need to examine whether self‐directed PARO use could be beneficial to older adults with dementia. We hypothesized that internally motivated and increased interaction time with PARO could generate benefits similar to other facilitated PARO activities.
3. **Future directions**: Our study revealed that self‐directed PARO use could reduce caregiver burden. Moreover, it holds clinical significance in addressing behavioral and psychological symptoms of dementia. This highlights the opportunity for making PARO available to older adults with dementia to use on their own time.

In Japan, while no RCT specifically investigating PARO exists, studies have reported greater preferences for PARO compared to other stuffed animals,^14^ reduced behavioral and psychological symptoms of dementia (BPSD), and reduced feelings of caregiver burden.^15^, ^16^ BPSD includes a range of neuropsychiatric disturbances such as agitation, aggression, depression, and apathy.^17^ While PARO can be used in both facility care and home care, using PARO at home is argued to be beneficial in decreasing caregiver fatigue and improving communication.^16^ Group homes in Japan are designed to enable residents with dementia to live independently in small groups while receiving care and assistance, with a physical layout and social dynamics that may closely resemble a Japanese home. Hence, in this study, we investigated the effects of PARO in a group‐home setting and explored the impact of varying PARO intervention frequency. Based on the hypothesis that longer exposure time would reduce BPSD more and lower the honorific burden level, this research compared results across different exposure times: once a week and thrice a week, each for 1 h.

## 2 METHODS

### 2.1 Trial design, setting, and participants

We adopted a cluster‐randomized design, which randomized group allocation based on the facility (rather than individually). We compared two groups within 12 care units – a large area into which a group home is divided – for people with dementia in six group‐home facilities in Japan's Tokyo and Hyogo prefectures. The allocation ratio was 1:1 for the two groups. We partnered with certified dementia‐care (group home) facilities that met the “Standards for Personnel, Equipment, and Operation of Designated Community‐based Services” by the health and welfare ordinance.^18^ Staff members' cooperation was vital as they received a short training course on maintaining PARO.

Due to operational constraints, this study lasted only a month, whereas previous studies often spanned 5 weeks to 3 months. Given the many studies supporting the effectiveness of PARO, we did not include a control group without intervention. Instead, we focused on the different exposure times and their impact, hence comparing a group that interacted with PARO once a week for 1 h and a group that interacted with PARO thrice a week for 1 h. The condition of thrice a week (the longer exposure time) was determined by considering the maximum capacity of the facility, with the acceptable range of one to three times a week, based on the opinion survey of the cooperating facilities.

Participant eligibility criteria were as follows: (1) individuals aged 65 years or older, (2) residents of a group home for people with dementia, (3) observed to be physically and mentally capable of spending time in the shared space, such as the ability to recognize and take PARO and roam and sit within the shared space, and (4) displayed no apparent aversion toward PARO. Consent for participation was obtained from the participants’ family members or legal guardians, who willingly signed the consent forms. To determine whether any participants had an aversion to PARO before the intervention, we asked facility staff whether any potential participants had an aversion to animals or plush toys. Then, for those who agreed to participate, the facility staff observed the participants' reactions when PARO was introduced, excluding those who displayed aversion to PARO from future sessions.

### 2.2 Selection, randomization, and blinding

The study started recruitment on February 15, 2019, and abruptly concluded on March 31, 2020. Letters of request were randomly sent to eligible group homes. Once a facility consented to participate in the research, recruitment leaflets were distributed to the residents' family members and legal guardians. Those who gave their consent were included in the study. Consent was confirmed verbally with the resident whenever possible. On evaluation and intervention days, the facility personnel confirmed in advance that the participants were in good health.

The randomization procedure was based on the number of floors in the facility. Each facility was categorized as one‐, two‐, or three‐floored. Each facility was then assigned to two groups. Upon receiving a notification of consent, the principal investigator contacted a research collaborator from a third‐party organization unaffiliated with any authors to randomly allocate the facility to either the once‐weekly or thrice‐weekly group using a computer‐generated random number table. Liaison personnel, independent of the allocator's organization, contacted the facility by phone and email to inform their assigned group. The allocation process was blinded (single‐blind) to research team members and collaborators (duly licensed occupational therapists) assigned to conduct assessments. The occupational therapist who oversaw the evaluation reviewed the collected data thoroughly before it was compiled and sent to data entry staff via mail. Statistical analysis of the results was commissioned to a statistical consultant.

PARO measures 57 cm in length, weighs 2.55 kg, and is designed for use in facilities with strict hygiene management. Equipped with artificial intelligence and various sensors, it responds to interactions such as verbal communication, gentle patting, and stroking by moving its front and rear legs, tilting its head, blinking, and making vocalizations – displaying cute, lifelike pet‐like behaviors. Through its artificial intelligence capabilities, PARO learns the names and positive words given to it. As it is used, it gradually adopts more affectionate gestures and vocalizations toward its user. PARO is rechargeable, and you can also interact with it while it's connected to the charging cable. However, once fully charged, users can place it on a table or on their lap, pick it up, and interact with it.

### 2.3 Intervention

The facility personnel were asked to place PARO at the center table in the day room (common room), where the residents often gathered according to the assigned time. One PARO unit was assigned to every four to five research participants. The facility personnel would announce “PARO is here” and “Please play with PARO” to encourage interaction. The facility personnel were instructed to assist only in getting to PARO, as required, and to avoid facilitating or being involved in any other active engagement. If someone were to monopolize PARO, we requested that facility personnel intervene to ensure all participants could interact. To verify the situation, the audio recorded by the integrated circuit recorder was used, as camera use was not permitted. After the 1‐h session, the facility personnel would announce, “It is time to put away PARO,” and remove PARO from the room. Considering the practical timeframe available at the facility, one session was set for 1 h.

Although the specific days for the intervention varied across facilities, it consistently lasted 1 h in the afternoon, between lunch and the afternoon snack (between 12 and 3 p.m.). The principal investigator, the occupational therapist in charge of the evaluation, and the facility personnel collaborated to determine the exact locations for the intervention and evaluation.

### 2.4 Outcome measures

To gather essential demographic information, the assigned research collaborator responsible for evaluation obtained data from the facility personnel. These data encompassed key details such as age, gender, diagnosis, level of care required, and level of independence in activities of daily living (ADLs).[Fig alz71163-fig-0002]

This study utilized the Neuropsychiatric Inventory Brief Questionnaire (NPI‐Q) as the primary outcome measure to assess the severity of BPSD and the caregiver's burden of care. The NPI‐Q, used widely worldwide, was developed by Kaufer and colleagues^19^ and cross‐validated by Cummings et al.^20^ It has a standardized Japanese version established by Matsumoto et al.^21^ The NPI‐Q comprises 12 items, including delusions, hallucinations, agitation/aggression, depression, anxiety, elation/euphoria, apathy/indifference, disinhibition, irritability, aberrant motor behavior, sleep and nighttime behavior disorders, and appetite and eating disorders. These items are scored in severity on a scale of 1 to 3 and burden of care on a scale of 0 to 5. Caregivers (informants) were provided with a paper‐based questionnaire and instructed to circle the relevant items based on their observations.

To ensure accurate evaluation and scoring of the NPI‐Q, the occupational therapist in charge of evaluation provided instructions to the chief care worker, who recorded the results. The occupational therapist in charge meticulously reviewed all records to confirm there were no omissions. Pre‐initiation records, collected before the intervention commenced, were made inaccessible during the evaluation session to maintain the integrity of the evaluation process.

The Mini‐Mental State Examination (MMSE), a widely used cognitive assessment tool, was also used.^22^ The MMSE evaluates cognitive function through a 30‐point test consisting of 11 questions that assess five areas: orientation, registration, attention and calculation, recall, and language. A score of 23 points or lower on the MMSE is considered indicative of dementia, with a sensitivity of 81% and specificity of 89%.^23^ The occupational therapist in charge of evaluation administered the MMSE concurrently with the collection of participants' basic information. This assessment, which took 6 to 10 min to complete, provided valuable additional insights into cognitive functioning.

### 2.5 Statistical method

To determine the appropriate sample size for this study, we conducted a pilot study in a group home that was excluded from the results section of this article. The pilot study involved 10 participants, and NPI‐Q severity scores were collected before and after a 1‐month intervention. The average between‐group difference in the amount of change was 1.04, with a standard deviation (SD) of 2.08 and 2.14 for each group. Considering a 10% dropout rate, we aimed for a sample size of 147 participants. The analysis method employed a linear mixed model with fixed effects: Time [Before, After], Group [Once weekly, Thrice weekly], and Interaction [Time*Group] and random effects: Facility and Participant [Facility nested]. To account for the clustering, participants were nested within their respective facilities as a variable effect. Estimated means and 95% confidence intervals were calculated for the pre‐intervention values, post‐intervention values, and their changes. Additionally, estimated means and 95% confidence intervals were computed to examine the difference in change between the two groups. Statistical analysis was performed using SPSS 24.0 for Windows (IBM Japan). The significance level was set at 5% two‐sided. Subgroup analyses were not performed.

### 2.6 Ethical considerations

This study was conducted with the approval of the Ethics Review Board (Ethics No. 17094). It was conducted in compliance with the Helsinki Convention and with the consent of the participants’ family members. The participants were verbally consulted to participate in the activities, and the final decision regarding the participants was made after several facility personnel confirmed their physical condition and willingness to participate in the activities on the day of the intervention. This research is registered under the UMIN Clinical Trials Registry (UMIN‐CTR: UMIN000039473).

## 3 RESULTS

**FIGURE 2 alz71163-fig-0002:**
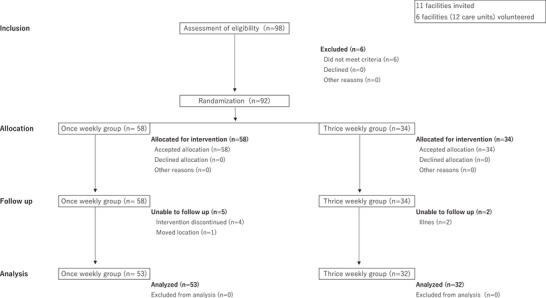
Legend: Process from recruitment to study completion. Eleven facilities were invited, six facilities (including 12 units) volunteered, 98 candidates were recruited, and 92 participated in the study. Fifty‐eight were assigned to the once‐weekly group and 34 to the thrice‐weekly group. The once‐weekly group had 5 dropouts, and 2 dropped out of the thrice‐weekly group, making a final total of 85 eligible for analysis.

Six of the initial 98 individuals who agreed to participate did not meet the criteria and were excluded. This left a total of 92 participants eligible for randomization. Among them, 58 participants were assigned to the once‐weekly group and 34 to the thrice‐weekly group. Throughout the study, five participants from the once‐weekly group and two participants from the thrice‐weekly group discontinued the intervention. As a result, the final analysis included 53 participants from the once‐weekly group and 32 participants from the thrice‐weekly group (Figure 2). The original sample size calculation projected a power of 80%, but because the trial was terminated early, the resulting sample sizes of 32 and 53 cases yielded a calculated power of 59%. Table 1 shows the baseline demographic and clinical characteristics for each group. Due to the emergence of COVID‐19 cases in Japan in January 2020, the study was prematurely concluded in March 2020. This decision was made to prioritize the health and safety of the study participants and relevant personnel.

**TABLE 1:** Baseline demographic and clinical characteristics for each group.

	Once‐weekly group (*n* = 53)	Thrice‐weekly group (*n* = 32)
**Sex**		
Female	44	31
Male	9	1
**Age**		
Youngest	65	78
Oldest	99	98
Average	86.8	87.8
Standard deviation	7.67	4.92
**Care level**		
Care level 1	7	6
Care level 2	9	10
Care level 3	20	11
Care level 4	6	2
Care level 5	11	3
**Level of independence in daily living for older adults with dementia**		
Ia	0	0
Ib	0	1
IIa	3	0
IIb	17	8
IIIa	11	11
IIIb	9	6
IV	12	6
M	1	0
**Independence in daily living of the elderly with disabilities**		
J1	0	0
J2	6	2
A1	17	15
A2	11	9
B1	4	4
B2	10	2
C1	1	0
C2	4	0
**Diagnosis**		
Alzheimer's disease	19	23
Juvenile‐onset Alzheimer's disease	1	0
Vascular dementia	3	0
Lewy body dementia	0	2
Frontotemporal dementia	1	0
Dementia	26	5
Not known	3	2

**FIGURE 3 alz71163-fig-0003:**
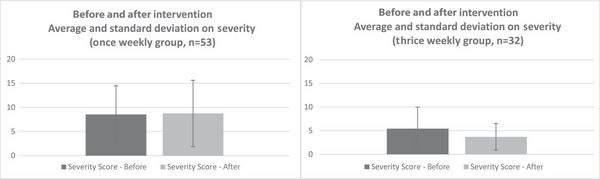
Legend: Average and standard deviation on severity, before and after intervention. The upper side of the bar chart shows the mean, and the length of the thin line extending up and down from the mean shows the standard deviation. For severity, the difference between the before and after was larger in the thrice‐weekly group than in the once‐weekly group, but it was not statistically significant.

Regarding the change in severity (Figure 3), the mean severity score in the once‐weekly group was 8.53 (SD = 5.95) at baseline, and after the intervention, it was 8.75 (SD = 6.87). For the thrice‐weekly group, the baseline mean severity score was 5.44 (SD = 4.57), and after the intervention, it was 3.69 (SD = 2.81). According to the analysis, there was no statistically significant difference in the “NPI‐Q severity score” between groups (adjusted least squares [LS] mean: −1.98, 95% CI: [−4.10, 0.15], *p* = 0.068).[Fig alz71163-fig-0003], [Fig alz71163-fig-0004]

**FIGURE 4 alz71163-fig-0004:**
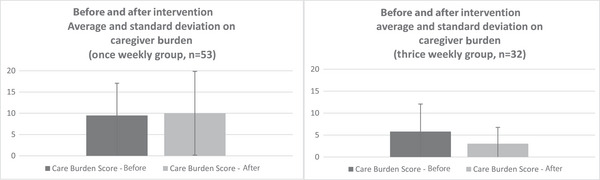
Legend: Average and standard deviation of caregiver burden, before and after intervention. The upper side of the bar chart shows the mean, and the length of the thin line extending up and down from the mean shows the standard deviation. The difference between the before and after was larger for the thrice‐weekly group than for the once‐weekly group, and a statistically significant difference was found.

The caregiver burden scores showed the following changes: In the once‐weekly group, the baseline mean was 9.49 (SD = 7.58), and after the intervention, the mean was 10.0 (SD = 9.86). In the thrice‐weekly group, the baseline mean was 5.81 (SD = 6.25), and after the intervention, the mean was 3.03 (SD = 3.74). The “NPI‐Q burden score” showed statistically significant improvement in the thrice‐weekly group compared to the once‐weekly group (LS mean: −3.29, 95% CI = [−6.26, ‐0.32], *p* = 0.030) (Figure 4).

Table 2 presents the results of the analysis comparing pre‐ and post‐intervention outcomes between the once‐weekly and thrice‐weekly groups. Sensitivity analysis, adjusting for gender, age, and care level, showed similar findings in severity and caregiver burden, thereby confirming the robustness of the results. No harm or unintended effects were reported in each group throughout the intervention.

**TABLE 2 alz71163-tbl-0002:** Change in outcome measure, before‐ and after‐intervention comparison between groups (once‐weekly vs thrice‐weekly).

	Group A: Once‐weekly (*n*=53)			Group B: Thrice‐weekly (*n*=32)			**Difference (Group B—A)**		
	LS mean	95% CI	*p* value	LS mean	95% CI	*p* value	**LS mean**	**95% CI**	***p* value**
NPI severity score									
Before	8.428	5.916, 10.940	–	5.487	2.013, 8.961	–			
After	8.654	6.142, 11.166	–	3.737	0.263, 7.211	–			
Amount of change	0.226	−1.076, 1.528	0.730	−1.750	−3.426, −0.074	0.041	**−1.976**	**−4.099, 0.146**	**0.068**
NPI Caregiver burden score									
Before	9.324	5.641, 13.006		5.818	0.712, 10.925				
After	9.833	6.150, 13.516		3.037	−2.070, 8.144				
Amount of change	0.509	−1.312, 2.331	0.579	−2.781	−5.125, −0.437	0.021	**−3.291**	**−6.259, −0.322**	**0.030**

Abbreviation: LS, least squares; NPI, Neuropsychiatric Inventory.

## 4 DISCUSSION

This study was conducted in a group home setting with minimal involvement of care workers, aiming to investigate the impact of varying levels of PARO exposure on the severity of BPSD and caregiver burden. The findings suggest that using PARO three times a week for 1 month can effectively reduce caregiver burden compared to once a week. PARO has a direct and indirect positive effect on caregivers.^16^ In this study, we observed a reduction in caregiver burden after 1 month of thrice‐weekly PARO use. Factors including walking around, aggression, screaming, low ADL levels, dysphoria/depression, disinhibition, restlessness, and subjective mood contribute to caregiver burden.^24^, ^25^ It is plausible that the reduced caregiver burden observed in this study is associated with the improvements in these BPSD. Moyle et al.^6^ conducted an RCT with minimal involvement of caregivers, demonstrating that interaction with a robot improved agitation, one of the BPSD symptoms in people with dementia. Our study showed a significant effect on a new outcome – reducing caregiver burden – under additional settings: a Japanese group‐home environment and a shorter intervention period. Furthermore, higher frequency use (thrice per week) was associated with this reduction.

In this study, the sample size was calculated assuming a power of 80% beforehand; however, the actual sample collected fell short, resulting in a power of approximately 59%. Therefore, caution is required when interpreting the results. This lack of statistical power likely caused the failure to detect a statistically significant difference in the primary outcome, BPSD severity. However, the observed LS mean difference (−1.98) in the thrice‐weekly group indicates a clinically meaningful trend toward improvement, consistent with results from previous studies. We think a significant effect could have been confirmed with a larger sample size. However, sensitivity analyses accounting for gender and disease severity also showed trends consistent with the primary analysis, suggesting that the results of this study are robust. Further analysis, particularly of the NPI‐Q subitems not examined in this study due to the focus on severity scores, would provide additional insights into the effects of PARO on other BPSDs. Additionally, considering the relatively wide confidence intervals observed, conducting a more detailed analysis of the sub‐items could be beneficial in future research.[Table alz71163-tbl-0002]

Further research is also required to investigate the sequential relationship of PARO's effect on individuals with dementia and their caregivers, as well as the duration and frequency of use. There was little change in severity score for the once‐weekly group, while there was a trend toward improvement for the thrice‐weekly group. No statistically significant difference was found in between‐group comparisons. However, since a LS mean difference of −1.98 is clinically meaningful, it is possible that the lack of statistical significance was due to an insufficient number of cases. The initial target sample size of 147 participants could not be achieved due to the COVID‐19 pandemic, possibly affecting the study's statistical power.

Previous studies reported that intervention periods ranging from 5 weeks to 3 months have shown effectiveness in addressing BPSD.^6^ Although our intervention period was 1 month, shorter than in previous studies, this duration was set based on realistic operational conditions in Japanese group homes. Even in such a brief period, PARO still yielded a significant reduction in caregiver burden, suggesting that PARO delivers rapid effects and is feasible for practical implementation.

This study aimed to examine the effects of robots while minimizing human influence as much as possible. However, previous research repeatedly pointed out that caregiver involvement is a key factor in enhancing the effectiveness of robots. Hensel et al.^26^ stated that collaboration with caregivers was essential for the use of robots in the home. Furthermore, Ghafurian et al.^27^ reported that the effectiveness of robots depended significantly on the relationship with caregivers and the social context. While robots contribute to supporting the independence of older adults and promoting social interaction, caregiver involvement enhances these effects. This indicates that human collaboration is a crucial element, rather than merely providing technical support. In actual care settings, it is believed that technology can be used more effectively with caregiver support. Therefore, it is crucial to develop and provide comprehensive caregiver training programs before implementing such systems. In the future, conducting long‐term research to assess the intervention's effectiveness in Japan's context would provide valuable insights.

### 4.1 Limitations and future directions

One notable limitation of this study was the COVID‐19 pandemic, which disrupted the research and led to its premature discontinuation. Our research team completed data collection from the first through sixth facilities before the nationwide outbreak. We did not proceed with data collection from the seventh facility onward due to government restrictions and used only the data already collected. Consequently, we consider that the pandemic had no impact on data quality. The reduced number of cooperating facilities limited the pool of potential participants and affected the generalizability of the findings. The reduced sample size due to pandemic‐related constraints underscores the need for caution when interpreting and generalizing the results. This has also prevented any opportunities to conduct follow‐up evaluations, which would be interesting to see whether results were maintained over time. Another possibility is that limited facility availability may have introduced selection bias, as the remaining facilities may differ in characteristics or demographics from the initially planned sample. Finally, leaving participants free to use PARO at will does not return an objective, measurable measure of how much they used it. Since this was an exploratory study, we included not only participants' direct interaction with the robot but also behaviors such as being present and enjoying watching it. However, to more accurately verify the effects of direct interaction, it is necessary to introduce highly objective methods, such as measuring usage time using cameras and sensors, to ensure accuracy. Nonetheless, this highlights the importance of internal motivation to benefit from using PARO.

Future research should consider these limitations and replicate the study under more optimal conditions to validate the findings. Although we have used a linear mixed model to account for the difference between groups, increased statistical similarity at baseline for allocating groups would be beneficial.

In this study, we aimed to capture the phenomenon comprehensively and selected the NPI‐Q as the primary outcome measure among standardized Japanese instruments. However, future studies should employ outcomes more suited to the actual situation by conducting secondary analyses of the present results. The inclusion of additional outcome measures and a wider variety of demographic data would also enrich the discussion. Future research could, therefore, employ a more appropriate sample size and study duration (3 months or more, as in previous studies), keep the same structure, balance the groups better, and expand the assessments and outcomes used to get a more complete view of the impact of PARO on this type of population. Finally, the challenges we faced should not discourage future investigations from exploring the potential benefits of PARO (and similar interventions) across different settings, as the findings from this study provide valuable insights into the potential effectiveness of such interventions in reducing caregiver burden and improving the well‐being of individuals with dementia.

## 5 CONCLUSION

In summary, this study tested the effects of PARO alone, without active encouragement from care workers trained in PARO techniques, and showed that, under minimal human influence, 1 month of PARO use could effectively reduce the burden on caregivers. Additionally, we hypothesize that the observed reduction in caregiver burden may have a positive ripple effect on the quality of care provided to individuals with dementia. Although this study did not directly measure the impact of caregiver burden on BPSD, it is reasonable to speculate that improved caregiver well‐being could contribute to better management and outcomes for BPSD. When caregivers experience a decreased sense of burden, they may have more energy, patience, and emotional well‐being to devote to their caregiving responsibilities. This change in well‐being has the potential to positively influence the BPSDs exhibited by the individuals under their care. Further research is needed to explore these relationships more comprehensively.

## CONFLICTS OF INTEREST STATEMENT

Takanori Shibata, the developer of PARO, is also the technical director of Intelligent System Co., Ltd. Shibata was not involved in the site selection, data acquisition, or analysis. Author disclosures are available in the Supporting Information

Chiyomi Yatsu, a professional translator, was compensated from the study's research funds for translation and English‐language editing of the manuscript and also contributed to the discussion section.

## CONSENT STATEMENT

All human participants provided informed consent.

## Supporting information

Supporting Information
